# Mortality Trends Among Early Adults in Germany, 2011 to 2023

**DOI:** 10.1001/jamanetworkopen.2025.37349

**Published:** 2025-10-14

**Authors:** Christof Kuhbandner, Matthias Reitzner

**Affiliations:** 1 Department of Human Sciences, University of Regensburg, Regensburg Germany; 2 Institute for Mathematics, Osnabrück University, Osnabrück, Germany

## Abstract

This cohort study compares mortality trends among adults aged 25 to 44 years in the US and Germany.

## Introduction

A recent study found that mortality among early adults (aged 25 to 44 years) in the US increased substantially since 2010, following a 2-phase pattern.^1^ The first phase, through 2019, was characterized by a steady rise in excess mortality, which then accelerated markedly during the COVID-19 pandemic and remained elevated thereafter. This increase was driven by external and natural causes, with drug-, alcohol-, transport-, homicide-, and COVID-19–related deaths accounting for the largest share of excess mortality. This study examines whether a similar pattern occurred among early adults in Germany.

## Methods

According to the German Research Foundation, this cross-sectional study was exempt from review and informed consent because no human participants were involved. We followed the STROBE reporting guideline.

Annual all-cause mortality probabilities for adults aged 25 to 44 years were calculated for the years 2000 to 2023 using the life table of the German Federal Statistical Office (GFSO). All-cause mortality was assigned to specific causes of death using mutually exclusive and exhaustive cause-specific death counts from the GFSO, aligned with the categories used in a previous US study (eTable in Supplement 1).^1^ Expected and excess deaths in the years 2011 to 2023 were modeled using exponential mortality trend functions and linear mortality trends, based on the 2000 to 2010 baseline (eMethods in Supplement 1). Data were analyzed from April to July 2025 using LibreOffice Calc version 7.3.7.2 (Document Foundation).

## Results

From 2000 to 2023, 431 920 deaths occurred among individuals aged 25 to 44 years in Germany. In contrast to the US, no increase in all-cause mortality was observed in Germany from 2011 to 2019 compared with the extrapolation of the pre-2011 trend (Figure). As in the US, Germany experienced a notable rise in all-cause mortality during the COVID-19 pandemic (Figure). Mortality rates in the US were substantially higher than in Germany across nearly all causes (Table). The number of all-cause deaths per 100 000 population in the US was 1.8 times higher than in Germany in 2011 (149.5 vs 75.5), 2.4 times higher in 2019 (162.2 vs 66.3), and 2.8 times higher in 2023 (192.2 vs 69.1). The increase in excess deaths per 100 000 during the pandemic (2020 to 2022) relative to prepandemic years (2017 to 2019) was also more pronounced in the US (57.9 vs 3.74).

**Figure.**
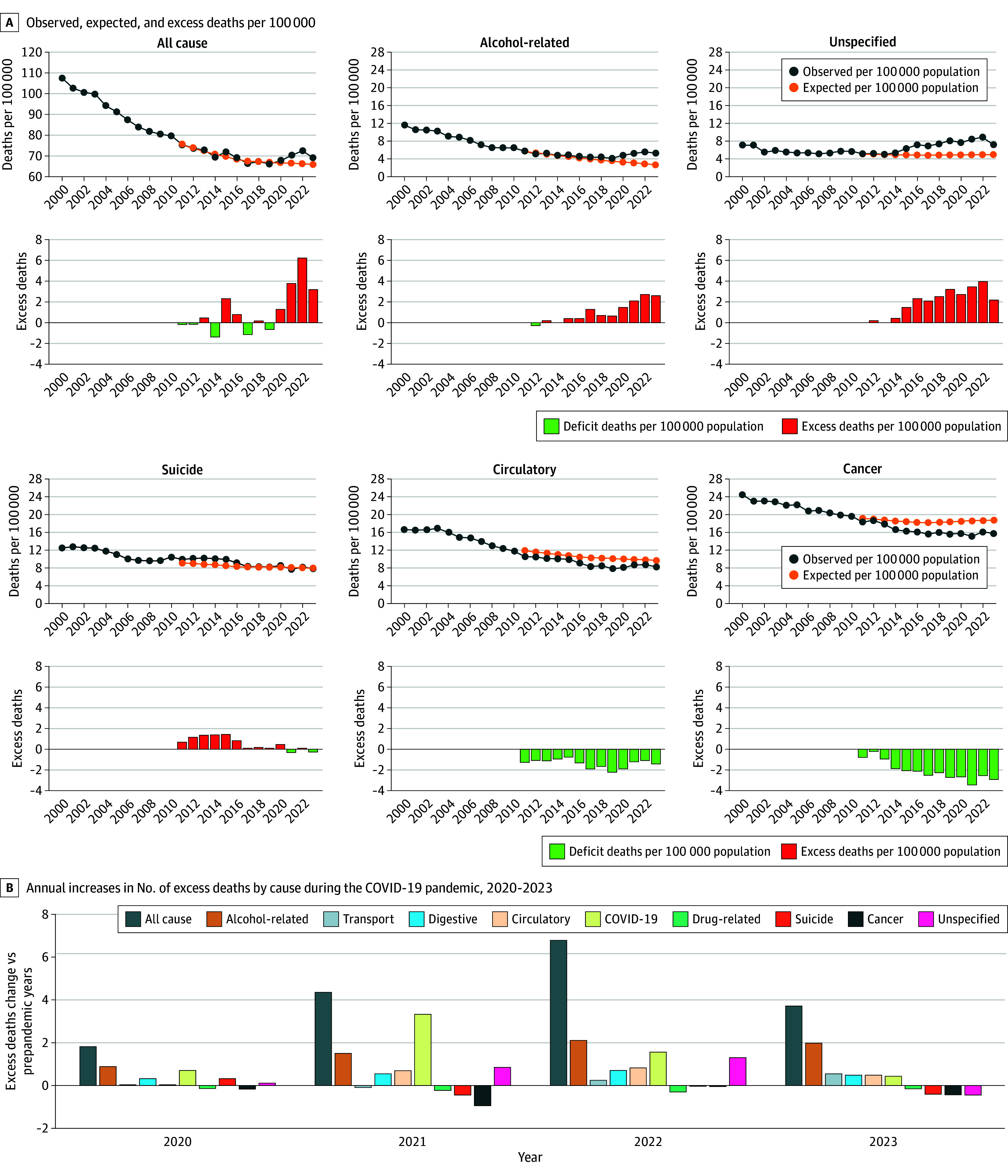
Observed, Expected, and Excess Deaths by Cause Among Adults Aged 25 to 44 Years in Germany, 2011 to 2023 A, The number of observed (blue dots), expected (orange dots), and excess deaths (red bars: excess mortality; green bars: mortality deficit) by year for all-cause mortality and the 5 leading causes of death in the 25 to 44 years age group. Observed and expected deaths are expressed per 100 000 population. Excess deaths are calculated as the difference between observed and expected mortality, with expected rates projected from baseline trends (2000 to 2010). B, Annual increases in the number of excess deaths by cause during the COVID-19 pandemic (2020 to 2023), relative to the prepandemic years (mean 2017 to 2019), for the 10 causes of death with the largest increases or decreases in excess mortality. In both panels, causes of death are listed in descending order according to the extent of excess mortality observed in 2023.

**Table.** Observed Deaths, Expected Deaths, and Excess Deaths per 100 000 Population by Cause of Death in Germany and the US, 2023

Cause of death^b^	Germany				US^a^		
	Deaths, No. per 100 000 population		Ratio of observed to expected	Excess deaths, No. per 100 000 population	Observed deaths, No. per 100 000 population	Ratio of observed to expected	Excess deaths, No. per 100 000 population
	Observed	Expected					
All cause^c^	69.12	65.98	1.05	3.14	192.18	1.70	79.11
Alcohol-related^d^	5.32	2.72	1.95	2.59	10.86	2.58	6.65
Unspecified	7.18	5.03	1.43	2.15	NA	NA	NA
Transport^c^	2.79	1.00	2.79	1.79	17.30	2.75	11.00
Accidental Poisoning	2.40	1.70	1.41	0.70	NA	NA	NA
Other external	4.13	3.54	1.17	0.59	6.89	1.02	0.12
Other natural	4.62	4.13	1.12	0.50	18.09	3.25	12.52
Digestive	1.90	1.44	1.32	0.46	4.76	1.34	1.20
COVID-19^c^	0.45	0.00	NA	0.45	0.85	NA	0.86
Respiratory^c^	1.72	1.55	1.11	0.18	4.22	1.01	0.06
Homicide^c^	0.43	0.41	1.06	0.02	11.41	2.29	6.42
Suicide^e^	7.72	8.02	0.96	−0.30	17.42	1.26	3.60
Endocrine^c^	2.17	2.61	0.83	−0.44	7.27	1.36	1.93
Nervous system	2.74	3.19	0.86	−0.45	NA	NA	NA
Drug-related^f^	1.43	2.18	0.66	−0.75	54.38	1.84	24.83
Circulatory^c^	8.26	9.72	0.85	−1.45	21.91	1.31	5.21
Cancer^c^	15.80	18.77	0.84	−2.96	16.84	1.29	3.78

Abbreviation: NA, not applicable.

^a^ US data are from the previously published study on mortality trends among early adults in the US.^1^

^b^ For detailed mapping of *International Statistical Classification of Diseases and Related Health Problems, Tenth Revision* (*ICD-10*) codes to cause-of-death categories, see the eTable in Supplement 1. Causes of death are ranked by excess mortality in 2023.

^c^ Identically coded cause-of-death categories in the German and US datasets.

^d^ Alcohol-related is more narrowly defined in the German dataset, as deaths due to accidental or intentional self-poisoning with alcohol are classified as accidental poisoning or suicide, not alcohol-related.

^e^ Suicide is more broadly defined in the German dataset, as it includes intentional self-poisonings with alcohol or drugs, which are classified separately as alcohol-related or drug-related in the US dataset.

^f^ Drug-related is more narrowly defined in the German dataset, as deaths due to accidental or intentional self-poisoning with drugs are classified as accidental poisoning or suicide, not drug-related.

In Germany, 4 causes—alcohol-related, circulatory system diseases, digestive system diseases, and unspecified causes—showed increases in excess mortality that mirrored the pattern of all-cause excess mortality during the pandemic. Combined, these causes accounted for most of the increase: 1.38 of 1.83 excess deaths per 100 000 population (75.8%) in 2020, 3.68 of 4.37 (84.3%) in 2021, 4.99 of 6.79 (73.6%) in 2022, and 2.53 of 3.72 (67.9%) in 2023. COVID-19 emerged as a new cause of death. This cause peaked in 2021, diverging from the pattern of all-cause excess mortality. Summing excess deaths for all 5 causes in 2021 yielded a total 1.6 times higher than the increase in all-cause excess deaths (7.03 vs 4.37 per 100 000 population). This discrepancy reflected an unexpected decline in mortality from several other causes that year and aligned with reassessments of deaths initially attributed to COVID-19, suggesting that a substantial proportion may not have been directly caused by COVID-19.^2,3^

## Discussion

This cross-sectional study found substantial divergences in early adult mortality trends between Germany and the US. Germany’s decline from 2000 to 2010 persisted through 2019, whereas US mortality rose over the same period. Both countries experienced elevated all-cause excess mortality during the COVID-19 pandemic, but increases were markedly greater in the US. Notably, causes with large US increases (eg, drug-related deaths, homicide, cancer) showed no excess or even reduced mortality in Germany. Conversely, others (eg, alcohol-related, circulatory, digestive deaths) had excess mortality in both countries, but larger in the US. This finding suggests that there was universal pandemic disruption to mortality in adults aged 25 to 44 years, with long-term consequences being country-specific for some causes but universal for others. Limitations of this study include the potential influence of changes in coding practices on cause-of-death trends, exemplified by rising unspecified deaths in Germany despite stable overall mortality from 2015 to 2019. Furthermore, estimates of expected deaths may vary with estimation methods, which should be considered when comparing mortality trends across studies.

## Notes

Supplement 1.
eMethods.
**eTable. **Cause-of-Death Categories and Associated *ICD-10* Codes

Supplement 2.
Data Sharing Statement
