# Supplementary material for: Mortality Trends Among Early Adults in Germany, 2011 to 2023
Source: JAMA Netw Open. 2025 Oct 14;8(10):e2537349. doi: 10.1001/jamanetworkopen.2025.37349 (PMC12522006; doi:10.1001/jamanetworkopen.2025.37349)
Supplement: Supplement 1. — eMethods. eTable. Cause-of-Death Categories and Associated ICD-10 Codes [file jamanetwopen-e2537349-s001.pdf]

Supplemental Online Content

Kuhbandner C, Reitzner M. Mortality trends among early adults in Germany, 2011 to 2023. *JAMA Netw Open*. 2025;8(10):e2537349.
doi:10.1001/jamanetworkopen.2025.37349

eMethods.
eTable. Cause-of-Death Categories and Associated *ICD-10* Codes

This supplemental material has been provided by the authors to give readers additional information about their work.

© 2025 Kuhbandner C et al. *JAMA Network Open*.

eMethods.

Data Sources

Cause-specific death counts: Cause-specific mortality data for the years 2000-2023 were obtained from the German Federal Statistical Office (Destatis) via its GENESIS-Online database.[1]

Population tables: Population tables for the years 2000-2023 were obtained from Destatis via its GENESIS-Online database.[2]

Causes of Death

The Federal Statistical Office codes deaths according to the International Statistical Classification of Diseases and Related Health Problems, 10th Revision (ICD-10). Based on physician-certified death certificates, the underlying cause of death is identified for each decedent and reported in aggregated cause-of-death categories, following the European Shortlist for Causes of Death.[3] The causes of death in the shortlist are organized in main categories (level 1 codes, e.g., cancer, diseases of the circulatory system, diseases of the respiratory system, external causes, etc.), and further in more detailed specific subcategories (level 2 and level 3 codes, e.g., alcohol abuse, suicide, transport accidents, etc.).

To enable comparison between Germany and the United States, cause-of-death categories were constructed based on this classification system that aligned as closely as possible with those used in the recent study on early mortality in the United States.[4] Table S1 shows the cause of death categories and associated ICD-10 codes used in the present study on mortality trends in Germany and in the recent study on mortality trends in the United States.

In the German dataset, from 2015 to 2019, the proportion of deaths coded as "unspecified" increased steadily from 7.8% to 12.3%, despite stable all-cause mortality. Correspondingly, the proportion of deaths attributed to circulatory diseases declined from 14.4% to 11.8%, and those attributed to suicide declined from 14.4% to 12.5%—a combined decrease that approximately offsets the increase in unspecified deaths. This near-equivalent redistribution in the context of stable overall mortality suggests changes in coding practices in Germany, such as stricter requirements for assigning specific causes in the absence of confirmatory evidence or updated guidance emphasizing the documentation of well-substantiated underlying causes.

Observed Deaths

For data protection reasons, since 2020 the Federal Statistical Office (Germany) has replaced the number of deaths with a dot in the cause-of-death statistics when only 1 or 2 cases occur in a given cause-of-death category within an age group. In these instances, the number of deaths was set to 1, and the remaining difference between the total number of reported all-cause deaths and the sum across all specific causes of death was assigned to the cause-of-death category 'Unspecified'.

Expected Deaths

The all-cause expected number of deaths for each year 2011-2023 was derived using population size and mortality probabilities for each sex and age separately. The population size was taken from population tables, and the mortality probabilities from the latest pre-pandemic life table 2017/19, both provided by the German Federal Statistical Office.[5] The calculations follow the standard actuarial approach, including exponential mortality trends provided by the German Actuarial Association,[6] and Farr's formula for taking into account the birthday problem, see Kuhbandner and Reitzner.[7]

The all-cause expected number of deaths is allocated to the various causes using the mortality statistics of the German Federal Statistical Office[1]. For each cause of death, the proportion of this cause of death to the total number of deaths was determined, including a linear trend function reflecting the most recent development. This is a slight simplification of the method suggested by Alai and colleagues.[8] Combining the total expected number of deaths with exponential mortality trend functions and linear mortality trends for different causes in the years 2000-2010 led to estimates for the expected number of deaths for each cause in the years 2011-2023. More details, estimates for the variance, and further references can be found in Kuhbandner & Reitzner.[7]

An analysis of the age distribution within the 25- to 44-year age group showed that it remained largely stable between 2000 and 2023, with only minor year-to-year variation. Accordingly, the original age structure was retained, allowing for the use of officially reported annual death counts without introducing uncertainty from synthetic age-standardization or redistribution procedures. This approach is consistent with that used in the recent study on early mortality in the United States.[4]

Excess Mortality

Cause-specific expected number of deaths are subtracted from the observed number of deaths for the years 2011-2023, yielding the absolute excess mortality, respectively absolute mortality deficit for each year and cause of death. The relative excess mortality is obtained as the ration between the absolute excess mortality and the expected number of deaths for this cause.

Further information available

All data and results for each cause of death are available at https://osf.io/pkxtg

Supplemental References

1. Federal Statistical Office of Germany (Statistisches Bundesamt). *GENESIS-Online database*. Table 23211-0002. Available at: https://www-genesis.destatis.de/datenbank/online/statistic/23211/table/23211-0002

2. Federal Statistical Office of Germany (Statistisches Bundesamt). *GENESIS-Online database*. Table 12411-0005. Available at: https://www-genesis.destatis.de/datenbank/online/statistic/12411/table/12411-0005

3. Eurostat. *Causes of Death Statistics Manual*. 2024 ed. Luxembourg: Publications Office of the European Union; 2024. Available at: https://ec.europa.eu/eurostat/web/products-manuals-and-guidelines/w/ks-gq-24-016

4. Wrigley-Field E, Raquib RV, Berry KM, Morris KJ, Stokes AC. Mortality trends among early adults in the United States, 1999-2023. JAMA Netw Open. 2025;8(1):e2457538. https://dx.doi.org/doi:10.1001/jamanetworkopen.2024.57538

5. Federal Statistical Office of Germany (Statistisches Bundesamt). GENESIS-Online database. Table 12621-0001. Available at: https://www-genesis.destatis.de/datenbank/online/statistic/12621/table/12621-0001

6. German Association of Actuaries (DAV): Life table DAV 2004R. Available at: https://aktuar.de/Dateien extern/DAV/LV/UT LV 7.pdf

© 2025 Kuhbandner C et al. *JAMA Network Open*.

7. Kuhbandner C, Reitzner M. Estimation of excess mortality in Germany during 2020–2022. *Cureus*. 2023;15(5):e39371. doi:10.7759/cureus.39371 https://dx.doi.org/doi:10.7759/cureus.39371

8. Alai DH, Arnold S, Sherris M. Modelling cause-of-death mortality and the impact of cause elimination. *Ann Actuar Sci*. 2015;9(1):167-186. https://dx.doi.org/10.1017/S174849951400027X

© 2025 Kuhbandner C et al. *JAMA Network Open*.

eTable. Cause-of-Death Categories and Associated *ICD-10* Codes

Cause of deaths	ICD-10 Codes (German data set)	ICD-10 Codes (US data set[4])
All Cause	A00–Y89	
Alcohol-related	F10, K70, K73–74	E24.4, F10, G31.2, G62.1, G72.1, I42.6, K70, K29.2, K85.2, K86.0, R78.0, X45, X65, Y15
Cancer	C00–D48	C00–D48
Circulatory	I00–I99	I00–I99 (excluding I42.6)
COVID-19	U07.1, U07.2	U07.1
Digestive	K00–K92 (excluding K70, K73–74)	K00–K92 (excluding K29.2, K85.2, K86.0)
Drug-related	F11-F16, F18-F19	X40–X44, X60–X64, X85, Y10–Y14 F11–F16, F19
Endocrine	E00–E88	E00–E88 (excluding E24.4)
Homicide	X85–Y09, Y87.1	X86–Y09, Y87.1
Nervous System	G00–G98	NA
Other external	W00–W99, X00–X48, X50-X59, Y10-Y98	W00–W99, X00–X39, X46–X59, Y16–Y36, Y40–Y84, Y86, Y87.2, Y88, Y89
Other natural	A00–A99, B00–B99, D50–D89, F01–F09, F20-F99, H00–H57, H60–L00–L98, M00–M99, N00–N98, O00–O99, P00–P96, Q00–Q99, R00–R95, U00–U99 (excluding U07.1, U07.2)	A00–A99, B00–B99, D50–D89, F01–F09, F17, F18, F20-F99, G00–G98 (excluding G31.2, G62.1, G72.1), H00–H57, H60–H93, L00–L98, M00–M99, N00–N98, O00–O99, P00–P96, Q00–Q99, R00–R99 (excluding R78.0), U00–U99
Poisoning (accidental)	X40–X49	NA
Respiratory	J00–J98	J00–J98
Suicide	X60–X84, Y87.0	X66–X84, Y87.0
Transport	V01–V99, Y85	V01–V99, Y85
Unspecified	R96–R99	NA

Abbreviations: NA.

© 2025 Kuhbandner C et al. *JAMA Network Open*.
