# Supplementary material for: Mortality Trends Among Early Adults in Germany, 2011 to 2023
Source: JAMA Netw Open. 2025 Oct 14;8(10):e2537349. doi: 10.1001/jamanetworkopen.2025.37349 (PMC12522006; doi:10.1001/jamanetworkopen.2025.37349)
Supplement: Supplement 2. — Data Sharing Statement [file jamanetwopen-e2537349-s002.pdf]

Data Sharing Statement

Kuhbandner. Mortality Trends Among Early Adults in Germany, 2011 to 2023. *JAMA Netw Open*. Published October 14, 2025. doi:10.1001/jamanetworkopen.2025.37349

Data
Data available: Yes
Data types: Data (not involving human participants)
How to access data: All data are available at https://osf.io/pkxtg
When available: With publication

Supporting Documents
Document types: None

Additional Information
Who can access the data: Anyone
Types of analyses: Any purpose
Mechanisms of data availability: Open internet (i.e., freely available)
